# Supplementary material for: Pharmacokinetics of a 503B outsourcing facility-produced theophylline in dogs
Source: PLoS One. 2022 Jan 6;17(1):e0262336. doi: 10.1371/journal.pone.0262336 (PMC8735617; doi:10.1371/journal.pone.0262336)
Supplement: S1 Table — Demographic data for individual dogs participating in study. Dogs with study IDs beginning with 1 underwent the IV phase followed by the PO phase. Dogs with study IDs beginning with 2 underwent the PO phase followed by the IV phase. Weight represents the dog’s weight at the study admission visit. FS = female spayed; MC = male castrated; MI = male intact. (PDF) [file pone.0262336.s001.pdf]

Study ID	Age (y)	Sex	Breed	Weight (kg)
1.1	4	FS	Labrador Retriever	27.3
1.2	5	MC	Boxer	27.5
1.3	6	MC	Rottweiler Mix	23.0
1.4	3	FS	Golden Retriever	25.9
2.1	6	MC	Standard Poodle	26.7
2.2	3	MI	Borzoi	36.5
2.3	2	FS	Standard Poodle	17.1
2.4	7	MC	Australian Cattle Dog	22.2
