# Supplementary material for: Pharmacokinetics of a 503B outsourcing facility-produced theophylline in dogs
Source: PLoS One. 2022 Jan 6;17(1):e0262336. doi: 10.1371/journal.pone.0262336 (PMC8735617; doi:10.1371/journal.pone.0262336)
Supplement: S2 Table — Plasma theophylline concentrations for individual dogs following a single intravenous dose of 11 mg/kg aminophylline. (PDF) [file pone.0262336.s002.pdf]

**Time (h)**	**Plasma Theophylline Concentration (μg/mL)**							
**Dog**	***1.1***	***1.2***	***1.3***	***1.4***	***2.1***	***2.2***	***2.3***	***2.4***
0	0	0	0	0	0	0	0	0
0.03	19.7	18.1	15.9	19.6	16.1	14.8	16.3	14.8
0.08	17.4	13.8	15.2	14.7	15.4	11.6	13.7	12.6
0.25	15.1	13.3	13.1	11.8	12.5	10.4	12.6	11.9
0.5	12.6	12.3	12.7	10.6	11.7	10	11.5	9.28
0.75	12.4	10.2	10	9.62	11.5	9.34	9.68	9.16
1	12.1	9.83	9.71	9.55	10.8	9.15	8.69	8.85
2	11.6	8.11	9.67	8.67	8.32	8.15	8.27	7.65
4	8.52	5.74	7.18	6.28	7.38	7.63	8.01	5.95
8	6.06	3.15	5.86	4.28	4.82	5.3	4.57	4.43
12	5.09	1.9	3.73	2.61	2.92	4.52	3.2	2.92
24	1.37	0.568	1.35	0.903	0.97	2.58	1.62	1.01
