# Supplementary material for: Pharmacokinetics of a 503B outsourcing facility-produced theophylline in dogs
Source: PLoS One. 2022 Jan 6;17(1):e0262336. doi: 10.1371/journal.pone.0262336 (PMC8735617; doi:10.1371/journal.pone.0262336)
Supplement: S3 Table — Plasma theophylline concentrations for individual dogs following a single oral dose of 10 mg/kg OFT. (PDF) [file pone.0262336.s003.pdf]

**Time (h)**	**Plasma Theophylline Concentration (μg/mL)**							
**Dog**	***1.1***	***1.2***	***1.3***	***1.4***	***2.1***	***2.2***	***2.3***	***2.4***
0	0	0	0	0	0	0	0	0
0.25	0	0.00589	0	0.00788	0	0	0	0
0.5	0	0.0172	0	0.0314	0.0155	0.0103	0	0
0.75	0	0.0417	0	0.116	0.0263	0.122	0.0282	0
1	0	0.16	0	0.185	0.0371	0.302	0.111	0
1.5	0	0.338	0.0206	0.327	0.116	1.35	0.277	0.0127
2	0	0.532	0.0312	0.639	0.14	1.85	0.397	0.754
4	0.224	3.87	0.567	0.999	0.377	5.36	1.61	5.69
8	5.85	6.41	4.06	5.29	4.16	7.97	4.16	6.23
12	8.01	4.25	6.79	7.76	6.73	7.25	7.1	5.34
18	4.88	2.02	5.85	3.78	4.18	4.7	5.42	2.57
24	3.22	0.93	3.37	2.42	2.86	3.67	3.28	1.61
48	0.447	0	0.381	0.448	0.281	1.17	0.841	0.211
