# Supplementary material for: Pharmacokinetics of a 503B outsourcing facility-produced theophylline in dogs
Source: PLoS One. 2022 Jan 6;17(1):e0262336. doi: 10.1371/journal.pone.0262336 (PMC8735617; doi:10.1371/journal.pone.0262336)
Supplement: S4 Table — Pharmacokinetic parameters from non-compartmental analysis of single dose intravenous aminophylline in individual dogs. The dosage listed is presented as theophylline equivalent. D = dosage; λz = terminal rate constant; t1/2 = terminal half-life; C0 = calculated concentration at time 0 for intravenous phase; AUCobs = observed area under the curve; AUC0-∞ = AUC extrapolated to infinity; AUC0-∞/D = AUC0-∞ normalized to dosage; AUC%Extrap = percent AUC extrapolated; Vz = apparent volume of distribution during terminal phase; Cl = clearance; AUMCobs = observed area under the moment curve; AUMC0-∞ = AUMC extrapolated to infinity; AUMC%Extrap = percent AUMC extrapolated; MRT = mean residence time. (PDF) [file pone.0262336.s004.pdf]

	Dog							
**Parameter**	***1.1***	***1.2***	***1.3***	***1.4***	***2.1***	***2.2***	***2.3***	***2.4***
D (mg/kg)	8.65	8.45	8.58	9.77	8.44	8.41	8.78	8.83
λ_ζ (η^{-1})	0.093	0.106	0.087	0.097	0.100	0.045	0.063	0.091
$t_{1/2}$ (h)	7.47	6.57	8.00	7.14	6.94	15.27	11.01	7.61
C_0 (mg/mL)	21.40	21.69	16.38	23.74	16.58	17.41	18.30	16.48
AUC_{obs} (mg*h/mL)	136.2	77.7	114.5	91.5	100.8	122.8	105.9	91.3
$AUC_{0-¥}$ (mg*h/mL)	150.9	83.1	130.1	100.9	110.6	179.7	131.6	102.4
$AUC_{0-¥}$/D (mg*h/mL)/(mg/kg)	17.4	9.8	15.2	10.3	13.1	21.4	15.0	11.6
$AUC_{\%Extrap}$ (%)	9.8	6.5	12.0	9.2	8.8	31.6	19.6	10.8
V_z (mL/kg)	617.9	962.4	762.0	998.4	764.0	1031.6	1059.7	946.6
Cl (mL/kg/h)	57.3	101.6	66.0	96.9	76.3	46.8	66.7	86.2
$AUMC_{obs}$ (mg*h^2/mL)	1029.3	468.6	865.5	628.8	698.9	1100.4	817.1	671.0
$AUMC_{0-¥}$ (mg*h^2/mL)	1542.7	648.7	1419.7	948.0	1029.2	3716.5	1843.6	1059.2
$AUMC_{\%Extrap}$ (%)	33.3	27.8	39.0	33.7	32.1	70.4	55.7	36.6
MRT (h)	10.22	7.80	10.91	9.40	9.31	20.68	14.01	10.34
