# Supplementary material for: Pharmacokinetics of a 503B outsourcing facility-produced theophylline in dogs
Source: PLoS One. 2022 Jan 6;17(1):e0262336. doi: 10.1371/journal.pone.0262336 (PMC8735617; doi:10.1371/journal.pone.0262336)
Supplement: S5 Table — Pharmacokinetic parameters from non-compartmental analysis of single dose oral OFT in individual dogs. D = dosage; λz = terminal rate constant; t1/2 = terminal half-life; TMAX = time at maximum concentration; CMAX = maximum concentration; AUCobs = observed area under the curve; AUC0-∞ = AUC extrapolated to infinity; AUC0-∞/D = AUC0-∞ normalized to dosage; AUC%Extrap = percent AUC extrapolated; AUMCobs = observed area under the moment curve; AUMC0-∞ = AUMC extrapolated to infinity; AUMC%Extrap = percent AUMC extrapolated; MRT = mean residence time; MAT = mean absorption time; F = bioavailability. (PDF) [file pone.0262336.s005.pdf]

	**Dog**							
**Parameter**	***1.1***	***1.2***	***1.3***	***1.4***	***2.1***	***2.2***	***2.3***	***2.4***
D (mg/kg)	10.26	9.75	9.74	11.49	10.07	10.30	10.29	10.14
λ_{ζ} (η^{-1})	0.080	0.127	0.091	0.071	0.092	0.047	0.061	0.084
$t_{1/2}$ (h)	8.62	5.47	7.62	9.78	7.54	14.84	11.44	8.28
T_{MAX} (h)	12.00	8.00	12.00	12.00	12.00	8.00	12.00	8.00
C_{MAX} (mg/mL)	8.01	6.41	6.79	7.76	6.73	7.97	7.10	6.23
AUC_{obs} (mg*h/mL)	147.1	74.3	142.2	128.4	123.0	184.6	149.5	111.7
$AUC_{0\text{-}¥}$ (mg*h/mL)	152.6	81.7	146.3	134.7	126.1	209.7	163.4	114.3
$AUC_{0\text{-}¥}$/D (mg*h/mL)/(mg/kg)	14.9	8.4	15.0	11.7	12.5	20.4	15.9	11.3
$AUC_{\%Extrap}$ (%)	3.6	9.0	2.9	4.7	2.4	11.9	8.5	2.2
$AUMC_{obs}$ (mg*h^2/mL)	2614.2	793.3	2608.7	2186.3	2184.8	3262.9	2830.3	1568.6
$AUMC_{0\text{-}¥}$ (mg*h^2/mL)	2950.1	1027.6	2855.7	2579.0	2364.8	5001.0	3726.0	1719.7
$AUMC_{\%Extrap}$ (%)	11.4	22.8	8.7	15.2	7.6	34.8	24.0	8.8
MRT (h)	19.33	12.58	19.51	19.15	18.76	23.85	22.81	15.05
MAT (h)	9.11	4.78	8.60	9.75	9.45	3.17	8.80	4.71
F (%)	85	85	99	114	96	95	106	97
